# Mild Autonomous Cortisol Secretion Is Associated with Computed Tomography-Derived Hepatic Steatosis in Patients with Adrenal Incidentaloma

**DOI:** 10.3390/medicina62102010

**Published:** 2026-10-17

**Authors:** Samet Alkan, Mustafa Farasat, Can Akcura, Sedat Can Guney, Merve Buyuker, Gokhan Pekindil, Nilufer Ozdemir, Zeliha Hekimsoy

**Affiliations:** 1 Department of Endocrinology and Metabolic Diseases, Faculty of Medicine, Manisa Celal Bayar University, 45030 Manisa, Türkiye; 2 Department of Endocrinology and Metabolic Diseases, Izmir City Hospital, 35540 Izmir, Türkiye; 3 Department of Radiology, Faculty of Medicine, Manisa Celal Bayar University, 45030 Manisa, Türkiye; 4 Department of Endocrinology and Metabolic Diseases, Democracy University Buca Seyfi Demirsoy Education and Research Hospital, 35390 Izmir, Türkiye; 5 Department of Endocrinology and Metabolic Diseases, Bakircay University Cigli Education and Research Hospital, 35620 Izmir, Türkiye; 6 Department of Radiology, Sarikamis State Hospital, 36200 Kars, Türkiye

**Keywords:** adrenal incidentaloma, mild autonomous cortisol secretion, hepatic steatosis, computed tomography, urinary free cortisol, liver attenuation, MASLD

## Abstract

*Background and Objectives*: Mild autonomous cortisol secretion (MACS) has been associated with adverse metabolic outcomes, but its relationship with hepatic steatosis remains incompletely understood. This study aimed to compare computed tomography (CT)-derived hepatic steatosis parameters between patients with MACS and non-functioning adrenal incidentaloma (NFAI) and to evaluate the associations between biochemical markers of cortisol secretion and hepatic attenuation measures. *Materials and Methods*: This retrospective study comprised 123 patients with adrenal incidentaloma (39 MACS and 84 NFAI) who underwent unenhanced abdominal CT between January 2020 and November 2024. Hepatic steatosis was assessed using mean liver attenuation, liver–spleen attenuation difference, liver-to-spleen attenuation ratio, and conventional CT-defined steatosis thresholds. Non-invasive metabolic indices were also computed. Associations between cortisol secretion parameters and CT-derived hepatic attenuation measures were examined using correlation analysis and multivariable regression. *Results*: The liver-to-spleen attenuation ratio was lower in the MACS group, although this difference did not reach statistical significance (*p* = 0.050), and a significantly lower liver–spleen attenuation difference (*p* = 0.048) was observed compared with the NFAI group, whereas mean liver attenuation (*p* = 0.167), conventional CT-defined hepatic steatosis, and non-invasive metabolic indices did not differ significantly between groups. Among biochemical markers of cortisol secretion, only 24-h urinary free cortisol showed significant correlations with all CT-derived hepatic attenuation parameters. After adjustment for age, sex, and BMI, urinary free cortisol remained significantly associated with liver–spleen attenuation difference (*p* = 0.047), whereas its associations with liver attenuation (*p* = 0.056) and liver-to-spleen attenuation ratio (*p* = 0.051) did not reach statistical significance. *Conclusions*: Patients with MACS exhibit subtle CT-derived hepatic fat accumulation despite similar conventional CT-defined steatosis and non-invasive metabolic indices compared with patients with NFAI. Continuous CT-derived attenuation parameters may be more sensitive than conventional CT-defined steatosis thresholds for detecting subtle hepatic fat accumulation associated with mild cortisol excess. The consistent associations observed between 24-h urinary free cortisol and CT-derived hepatic attenuation parameters, including the significant association with liver–spleen attenuation difference after adjustment for age, sex, and BMI, warrant confirmation in larger prospective studies.

## 1. Introduction

The widespread use of cross-sectional imaging has made adrenal incidentalomas (AIs) an increasingly common finding in routine clinical practice. Most of these lesions are benign and non-functioning; however, a substantial proportion show mild autonomous cortisol secretion (MACS), previously termed subclinical Cushing’s syndrome (CS). MACS involves persistent, low-level autonomous cortisol excess without the overt clinical manifestations of CS [1]. Accumulating evidence links MACS to adverse cardiometabolic and musculoskeletal outcomes, including hypertension, impaired glucose metabolism, dyslipidemia, obesity, osteoporosis, sarcopenia, and a higher risk of cardiovascular events. These observations suggest that even modest but persistent cortisol excess may exert clinically relevant systemic effects [2,3,4].

Metabolic dysfunction-associated steatotic liver disease (MASLD), previously known as non-alcoholic fatty liver disease (NAFLD), ranks among the most prevalent chronic liver diseases globally. It is strongly associated with obesity, insulin resistance, and metabolic syndrome [5,6]. Experimental and clinical evidence indicates that glucocorticoid excess promotes hepatic lipid accumulation through multiple mechanisms, including enhanced adipose tissue lipolysis, increased hepatic free fatty acid influx, stimulation of de novo lipogenesis, and worsening insulin resistance. Accordingly, hepatic steatosis is a well-recognized metabolic complication of overt endogenous hypercortisolism, particularly in patients with Cushing syndrome [7,8]. Although overt hypercortisolism has a well-established association with hepatic steatosis, considerably less research has addressed the relationship between AI/MACS and MASLD [9,10,11].

Liver biopsy remains the reference method for diagnosing hepatic steatosis and quantifying its extent. However, its invasive nature, sampling variability, and limited feasibility preclude its routine use in clinical practice. Accordingly, non-invasive approaches such as ultrasonography, computed tomography (CT), magnetic resonance imaging, and techniques based on elastography have been introduced to evaluate hepatic steatosis [12]. Among these, unenhanced CT is a widely available and well-validated imaging modality that enables quantitative evaluation of hepatic fat accumulation using liver attenuation measurements and attenuation indices derived from the liver and spleen [13,14]. Recent studies have evaluated CT-derived hepatic attenuation parameters in patients with adrenal incidentalomas and MACS, demonstrating associations between cortisol autonomy and hepatic fat accumulation [9,10,11,15]. However, the relationships between multiple quantitative CT-derived hepatic attenuation parameters and different biochemical measures of cortisol secretion, particularly 24-h urinary free cortisol, remain less well characterized.

Therefore, the present study aimed to compare CT-derived hepatic steatosis parameters between patients with non-functioning adrenal incidentalomas and MACS using quantitative unenhanced CT measurements. We further investigated the relationships between CT-derived attenuation parameters, established metabolic steatosis indices, and biochemical markers of cortisol secretion, with particular emphasis on cumulative cortisol burden assessed by 24-h urinary free cortisol.

## 2. Materials and Methods

### 2.1. Study Design and Participants

**Figure 1:**
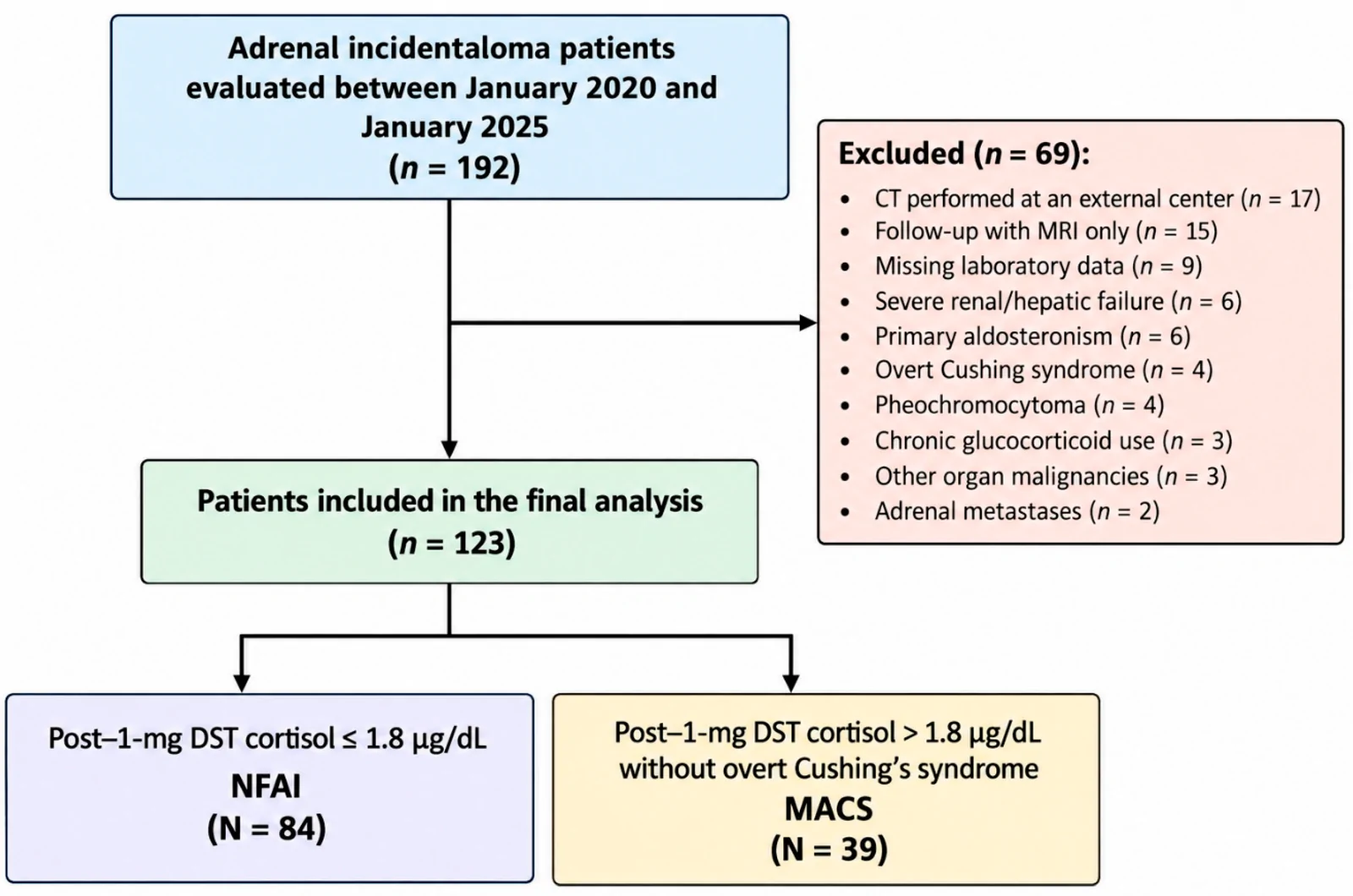
Flow diagram of study. Abbreviations: CT, computed tomography; DST, dexamethasone suppression test; MACS, mild autonomous cortisol secretion; MRI, magnetic resonance imaging; NFAI, non-functioning adrenal incidentaloma.

This single-center study used a retrospective design and was carried out at the endocrinology clinic of the Faculty of Medicine, Manisa Celal Bayar University. Institutional electronic records were reviewed retrospectively to identify consecutive adult patients assessed for adrenal incidentaloma in the endocrinology outpatient clinic from January 2020 through November 2024. Patients who underwent standard hormonal evaluation and unenhanced abdominal CT according to the institutional adrenal incidentaloma protocol were considered eligible for inclusion. Patients were excluded if they had overt Cushing syndrome, pheochromocytoma, primary aldosteronism, adrenocortical carcinoma, or metastases. Other reasons for exclusion were chronic glucocorticoid use, severe renal or hepatic failure, and incomplete clinical or imaging data. Included patients were subsequently classified as having NFAI or MACS. Figure 1 outlines the patient selection process.

Ethical approval was granted by the Ethics Committee of Manisa Celal Bayar University’s Faculty of Medicine on 27 November 2024 (Approval No. 20.478.426/2760). Written informed consent was not required, as the study involved retrospective evaluation of existing records.

### 2.2. Clinical and Hormonal Assessment

Patients included in the study had been routinely evaluated according to the institutional adrenal incidentaloma protocol. Initial hormonal assessment included a 1-mg overnight dexamethasone suppression test (DST) and measurement of 24-h urinary fractionated metanephrines. Patients with biochemical and clinical findings consistent with pheochromocytoma were excluded. Plasma aldosterone levels and renin activity were assessed in patients who had hypertension, hypokalemia, or both. Those with a positive aldosterone-to-renin ratio underwent confirmatory testing for primary aldosteronism and were excluded if the diagnosis was confirmed.

Patients whose serum cortisol exceeded 1.8 µg/dL after the DST were routinely admitted for further endocrine evaluation. This assessment included basal plasma ACTH, serum cortisol, 24-h urinary free cortisol (UFC), dehydroepiandrosterone sulfate (DHEA-S), and midnight serum cortisol measurements after two days of hospitalization. The 1-mg DST was subsequently repeated, and patients with persistent post-DST cortisol concentrations >1.8 µg/dL underwent an additional two-day 2-mg DST. Patients without clinical features of CS who demonstrated persistent post-dexamethasone serum cortisol concentrations >1.8 µg/dL together with biochemical evidence of ACTH-independent cortisol secretion were classified as having MACS. Patients were classified as NFAI if their cortisol level after the DST was ≤1.8 µg/dL [1].

Demographic characteristics, body mass index (BMI), clinical data, and routine biochemical parameters were retrospectively retrieved from the electronic medical records. These included fasting plasma glucose, fasting insulin, glycated hemoglobin (HbA1c), lipid profile, liver enzymes, platelet count, gamma-glutamyl transferase (GGT), creatinine, and other laboratory variables required for subsequent analyses.

### 2.3. CT Acquisition and Image Analysis

Abdominal CT scans without contrast were obtained for all patients with a 256-slice multidetector system (SOMATOM Drive, Siemens Healthineers, Forchheim, Germany). Images were acquired according to the institutional adrenal imaging protocol with a slice thickness of 1 mm. Adrenal incidentaloma was defined as an adrenal lesion measuring ≥1 cm in maximum diameter with imaging characteristics consistent with an adenoma. Patients with non-adenomatous adrenal lesions were excluded. For patients with multiple adenomas within the same adrenal gland, the largest lesion was considered for analysis. Lesion size, laterality (right, left, or bilateral), and unenhanced attenuation (Hounsfield units, HU) were recorded.

Two radiologists with expertise in abdominal imaging independently reviewed all CT scans without access to the patients’ clinical, biochemical, or hormonal information. Measurements were performed independently by both radiologists, and the final values were determined after consensus review.

**Figure 2:**
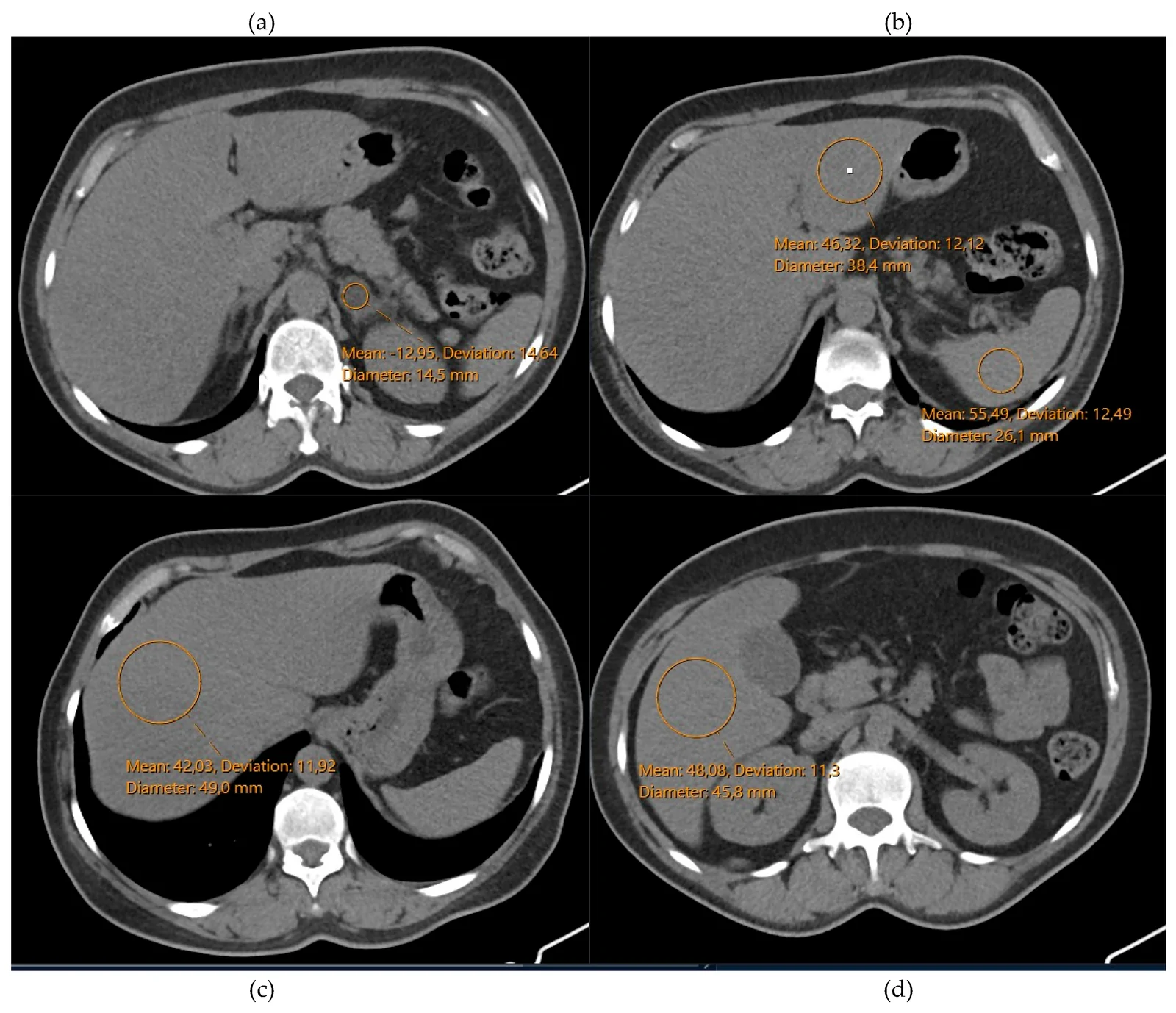
CT-based assessment of adrenal adenoma characteristics and hepatic attenuation. Representative unenhanced axial CT images illustrating the radiological measurements. (**a**) Adrenal adenoma attenuation measured using a circular region of interest (ROI) placed within the lesion, avoiding areas of necrosis, hemorrhage, calcification, and partial-volume effects. (**b**) Hepatic attenuation measured in segment II/III and splenic attenuation measured using a single ROI within homogeneous splenic parenchyma. (**c**) Hepatic attenuation measured in segment VIII. (**d**) Hepatic attenuation measured in segment V. Hepatic ROIs were placed avoiding major vessels, bile ducts, focal lesions, and imaging artifacts. Mean liver attenuation was calculated as the average attenuation of the three hepatic ROIs. The liver-to-spleen attenuation ratio was calculated by dividing mean liver attenuation by splenic attenuation, and the liver-spleen attenuation difference by subtracting splenic attenuation from mean liver attenuation. Abbreviations: CT, computed tomography; HU, Hounsfield unit; ROI, region of interest.

Hepatic attenuation was assessed by placing circular regions of interest (ROIs) within homogeneous liver parenchyma while carefully avoiding visible vessels, focal lesions, bile ducts, and imaging artifacts. ROIs were drawn as large as anatomically feasible to maximize representative sampling of the liver parenchyma. Three ROIs were obtained from Couinaud segments II/III, V, and VIII, and the mean liver attenuation was calculated as the average of these three measurements. Splenic attenuation was measured using a single large ROI placed within homogeneous splenic parenchyma while avoiding hilar vessels and focal lesions. The liver-to-spleen attenuation ratio (L/S ratio) and liver–spleen attenuation difference (L–S difference) were subsequently calculated from these measurements (Figure 2).

**Figure 3:**
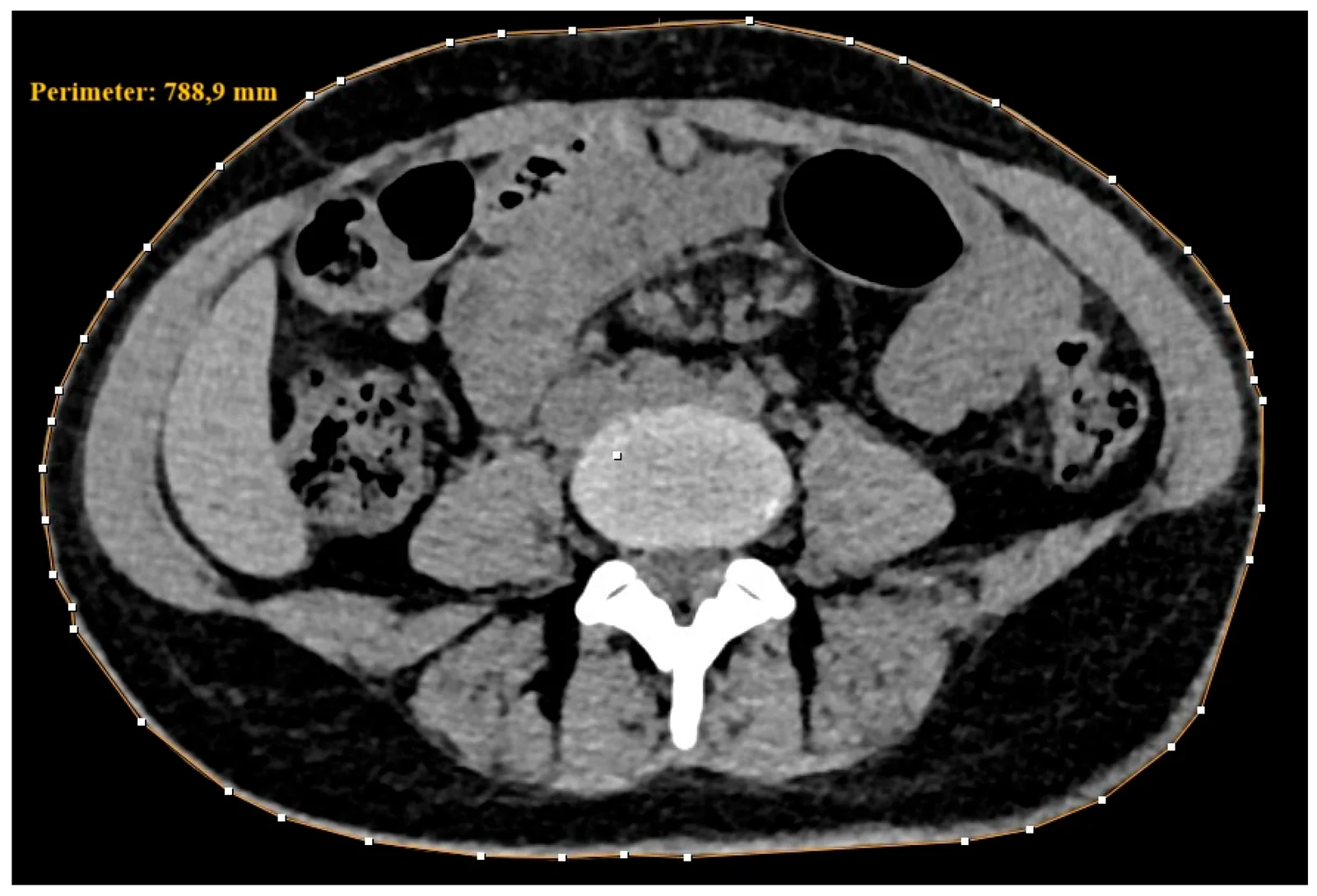
CT-derived waist circumference measurement. Representative axial CT image demonstrating waist circumference measurement at the L4–L5 intervertebral level. The outer body contour was manually traced, and the resulting perimeter was recorded as the CT-derived waist circumference. Abbreviation: CT, computed tomography.

CT-derived waist circumference was measured on axial images at the L4–L5 intervertebral level using a standardized technique (Figure 3). Circumference measurements were obtained only from CT examinations with complete visualization of the abdominal wall within the scan field of view. Cases with incomplete abdominal coverage precluding accurate circumference measurement were excluded from analyses involving CT-derived waist circumference [16].

### 2.4. Definitions and Calculation of Hepatic Steatosis and Metabolic Indices

Hepatic steatosis was evaluated using both continuous CT-derived attenuation parameters and conventional qualitative CT criteria. Continuous parameters included mean liver attenuation, liver-to-spleen attenuation ratio (L/S ratio), and liver–spleen attenuation difference (L–S difference). Conventional CT-defined hepatic steatosis was defined as liver attenuation <40 HU, an L/S ratio <1.0, or an L–S attenuation difference <5 HU, according to previously established criteria [12,13].

To further characterize metabolic status, insulin resistance, and liver-related risk, the triglyceride-glucose (TyG) index, TyG-waist-to-height ratio (TyG-WHtR), metabolic score for insulin resistance (METS-IR), fatty liver index (FLI), and fibrosis-4 (FIB-4) index were calculated according to previously validated equations [17,18,19,20,21]. CT-derived waist circumference was used to calculate the TyG-WHtR.

### 2.5. Statistical Analysis

Data were analyzed with IBM SPSS Statistics, version 22.0 (IBM Corp., Armonk, NY, USA). Normality of continuous data was evaluated with the Shapiro–Wilk test. For variables with a normal distribution, descriptive statistics were reported as means ± standard deviations (SDs), and between-group differences were tested with the independent-samples t-test. Variables that did not follow a normal distribution were summarized as medians with interquartile ranges (IQRs), with group comparisons based on the Mann–Whitney U test. Counts and percentages were used to describe categorical data; differences between groups were examined with Pearson’s χ^2^ or Fisher’s exact test, as appropriate.

To compare hepatic steatosis between study groups, both continuous CT-derived hepatic attenuation parameters and conventional categorical CT definitions of hepatic steatosis were analyzed. In addition, non-invasive metabolic indices, including the TyG index, TyG-WHtR, METS-IR, FLI, and FIB-4 index, were compared between groups.

Because cortisol secretion exhibits clinically meaningful variability primarily among patients with MACS, correlation analyses between cortisol secretion parameters and CT-derived hepatic steatosis measurements were performed exclusively within the MACS subgroup using Spearman’s rank correlation coefficients.

To further evaluate the independent associations between cortisol secretion and CT-derived hepatic steatosis, separate multivariable linear regression models were constructed for each CT-derived hepatic attenuation parameter (liver attenuation, liver-to-spleen attenuation ratio, and liver–spleen attenuation difference). Age, sex, body mass index (BMI), and 24-h urinary free cortisol were entered simultaneously into each model using the Enter method.

Two-sided tests were used throughout, with statistical significance defined as *p* < 0.05.

## 3. Results

### 3.1. Baseline Characteristics

The study population consisted of 123 patients with adrenal incidentalomas: 84 with NFAI and 39 with mild autonomous cortisol secretion (MACS). Table 1 presents their demographic, clinical, hormonal, and biochemical characteristics at baseline. The groups showed no significant differences in age, sex distribution, body mass index, hypertension, diabetes mellitus, fasting glucose, HbA1c, lipid parameters other than HDL cholesterol, liver enzymes, or platelet count. Dyslipidemia was more prevalent in the MACS group than in the NFAI group (35.9% vs. 19.0%, *p* = 0.043). The MACS group also had larger adrenal adenomas (30.5 ± 11.0 vs. 20.3 ± 8.3 mm, *p* < 0.001) and more frequent bilateral adrenal lesions (*p* = 0.042). Cortisol concentrations after the dexamethasone suppression test were higher, while basal ACTH levels were lower, in patients with MACS (both *p* ≤ 0.001). HDL cholesterol was also lower in the MACS group (54.4 ± 11.8 vs. 58.5 ± 12.4 mg/dL, *p* = 0.039).

### 3.2. CT-Derived Hepatic Steatosis Parameters and Non-Invasive Hepatic Steatosis/Fibrosis Indices

CT-derived hepatic steatosis parameters and non-invasive hepatic steatosis/fibrosis indices are presented in Table 2. Waist circumference measured on CT and splenic attenuation were comparable between the NFAI and MACS groups. Although median hepatic attenuation was lower in patients with MACS, the difference did not reach statistical significance (*p* = 0.167). In contrast, the liver–spleen attenuation difference was significantly lower in the MACS group than in the NFAI group (8.2 vs. 13.2 HU, *p* = 0.048), whereas the liver-to-spleen attenuation ratio was lower in the MACS group but did not reach statistical significance (1.15 vs. 1.24, *p* = 0.050).

When conventional CT diagnostic thresholds for hepatic steatosis were applied, the prevalence of hepatic steatosis showed no significant difference between the groups, regardless of the definition used (hepatic attenuation <40 HU, liver-to-spleen attenuation ratio <1.0, or liver–spleen attenuation difference <5 HU; all *p* > 0.05). The groups also showed no significant differences in non-invasive indices of hepatic steatosis or fibrosis, including the FIB-4 index, Fatty Liver Index, TyG index, waist-to-height ratio, TyG-WHtR, and METS-IR (all *p* > 0.05).

### 3.3. Correlations Between Cortisol Secretion Parameters and CT-Derived Hepatic Steatosis Parameters

Correlation analyses performed in the MACS subgroup are presented in Table 3. Post-dexamethasone suppression test cortisol, basal ACTH, and midnight cortisol concentrations were not significantly correlated with liver attenuation, liver-to-spleen attenuation ratio, or liver–spleen attenuation difference (all *p* > 0.05). In contrast, 24-h urinary free cortisol demonstrated significant inverse correlations with all three CT-derived hepatic steatosis parameters: liver attenuation (ρ = −0.427, *p* = 0.009), liver-to-spleen attenuation ratio (ρ = −0.370, *p* = 0.026), and liver–spleen attenuation difference (ρ = −0.396, *p* = 0.017).

### 3.4. Multivariable Linear Regression Analyses

To determine whether the observed associations were independent of age, sex, and body mass index, separate multivariable linear regression models were constructed for each CT-derived hepatic steatosis parameter (Table 4). Body mass index was independently associated with liver attenuation (standardized β = −0.365, *p* = 0.038), whereas age and sex were not significantly associated with any CT-derived hepatic steatosis parameter. After adjustment for age, sex, and BMI, 24-h urinary free cortisol remained significantly associated with liver–spleen attenuation difference (β = −0.403, *p* = 0.047), while its associations with liver attenuation (β = −0.362, *p* = 0.056) and liver-to-spleen attenuation ratio (β = −0.397, *p* = 0.051) did not reach statistical significance. None of the overall regression models reached statistical significance.

## 4. Discussion

In this study, patients with MACS showed a significantly lower liver–spleen attenuation difference and a lower liver-to-spleen attenuation ratio that did not reach statistical significance compared with those with NFAI, suggesting subtle differences in hepatic fat accumulation despite the absence of differences in conventional CT-defined hepatic steatosis or established metabolic steatosis indices. Furthermore, among patients with MACS, urinary free cortisol (UFC), but not other cortisol-axis parameters, showed a consistent unadjusted correlation with all three CT-derived hepatic steatosis metrics. After adjustment for age, sex, and BMI, UFC remained inversely associated with all three CT-derived parameters, with the association with liver–spleen attenuation difference reaching statistical significance, while the associations with liver attenuation and liver-to-spleen attenuation ratio were of borderline statistical significance. These findings suggest that quantitative CT-derived hepatic attenuation parameters may detect subtle hepatic changes related to chronic cortisol excess before conventional CT thresholds or metabolic indices become abnormal.

Our findings are broadly consistent with previous studies reporting an association between autonomous cortisol secretion and hepatic steatosis. In one of the earliest CT-based studies, Rockall et al. demonstrated reduced hepatic attenuation in patients with overt Cushing’s syndrome, suggesting that chronic glucocorticoid excess promotes hepatic fat accumulation [8]. More recently, Candemir et al. reported lower mean liver attenuation values in metabolically healthy patients with MACS compared with BMI-matched patients with NFAI and identified post-DST cortisol as an independent predictor of hepatic steatosis [9]. Papanastasiou et al. found no significant difference in the prevalence of NAFLD between patients with adrenal incidentalomas and matched controls; however, they reported higher insulin resistance indices in patients with adrenal incidentalomas and identified waist-to-hip ratio and triglyceride levels as independent determinants of liver–spleen attenuation difference [11]. Our results extend these observations by demonstrating that quantitative CT-derived attenuation parameters, particularly the liver-to-spleen ratio and liver–spleen attenuation difference, were able to detect subtle differences between MACS and NFAI, even in the absence of significant differences in conventional CT-defined steatosis or commonly used metabolic steatosis indices. The magnitude of the differences observed in our study was more modest than that reported by Candemir et al. and Yu et al. [9,15]. Candemir et al. evaluated a more selected population after extensive metabolic and clinical exclusions and compared age-, sex-, and BMI-matched MACS and NFAI groups, whereas Yu et al. examined a larger cohort that also included patients with overt Cushing’s syndrome. These differences in study populations and design may partly explain the stronger between-group differences reported in those studies. Importantly, our findings do not indicate a clear increase in established CT-defined hepatic steatosis in MACS; rather, the observed differences were subtle and were apparent mainly in continuous CT-derived attenuation measures. This distinction may explain why conventional steatosis thresholds did not differ between groups despite differences in liver–spleen attenuation measures.

An important finding of the present study is that significant between-group differences were observed only for continuous CT-derived attenuation parameters, whereas conventional CT-defined hepatic steatosis thresholds did not distinguish patients with MACS from those with NFAI. This observation suggests that quantitative attenuation measurements may be more sensitive for detecting early or mild hepatic fat accumulation associated with subtle cortisol excess than dichotomous CT definitions. Conventional thresholds such as liver attenuation <40 HU, liver-to-spleen ratio <1.0, or liver–spleen attenuation difference <5 HU were originally developed to identify established hepatic steatosis rather than subtle quantitative changes in hepatic fat content [12,13,14]. In contrast, continuous attenuation parameters preserve the full spectrum of attenuation values and may therefore better reflect early metabolic alterations before diagnostic thresholds are exceeded. Our findings are also consistent with the study by Candemir et al., who demonstrated reduced mean liver attenuation in metabolically healthy patients with MACS despite relatively preserved metabolic status [9]. Extending these observations, our results suggest that continuous CT-derived attenuation parameters may reveal subtle hepatic differences even when conventional CT-defined steatosis and non-invasive metabolic indices remain comparable between groups. Because splenic attenuation did not differ between groups, the observed differences in liver-to-spleen attenuation indices are likely to reflect differences in hepatic attenuation rather than variation in splenic attenuation.

Another noteworthy finding was that 24-h urinary free cortisol, but not post-DST cortisol, ACTH, or midnight serum cortisol, showed significant unadjusted correlations with all three CT-derived hepatic attenuation parameters in patients with MACS. After adjustment for age, sex, and BMI, UFC was inversely associated with liver–spleen attenuation difference (β = −0.403, *p* = 0.047), while the inverse associations with liver attenuation (β = −0.362, *p* = 0.056) and liver-to-spleen attenuation ratio (β = −0.397, *p* = 0.051) were of borderline statistical significance. These findings differ from those of Candemir et al., who identified post-DST cortisol as an independent determinant of mean liver attenuation [9]. In contrast, our results are in agreement with the recent study by Apaydin et al. in which UFC independently predicted hepatic steatosis assessed by controlled attenuation parameters in patients with endogenous Cushing’s syndrome after adjustment for metabolic confounders [7]. Although the underlying mechanisms remain uncertain, one possible explanation is the relatively narrow range of post-DST cortisol concentrations within the MACS subgroup, as all patients fulfilled the diagnostic criterion for MACS. In contrast, UFC exhibited greater interindividual variability and may therefore better reflect cumulative cortisol exposure, resulting in a closer relationship with gradually developing hepatic fat accumulation than a single post-DST measurement. These findings are also consistent with the concept that different cortisol tests may capture distinct aspects of hypercortisolism and therefore differ in their ability to predict end-organ complications. Nevertheless, given the cross-sectional design of both studies, this hypothesis should be interpreted cautiously and requires confirmation in prospective longitudinal studies.

Despite the observed differences in CT-derived hepatic attenuation parameters, no significant between-group differences were found in TyG, TyG-WHtR, METS-IR, FLI, or FIB-4. This finding may reflect the relatively similar metabolic profiles of the MACS and NFAI groups, particularly with respect to BMI, glycemic status, and lipid parameters, which are key components of these composite indices. Moreover, these scores were primarily developed to identify hepatic steatosis, insulin resistance, or liver fibrosis in populations with overt metabolic dysfunction rather than subtle endocrine-mediated metabolic alterations. In particular, FIB-4 primarily reflects the risk of liver fibrosis rather than hepatic steatosis [20,21]. In addition, the relatively limited variability of these indices within our cohort may have reduced their ability to demonstrate between-group differences. Therefore, the absence of significant differences does not necessarily contradict our imaging findings. Instead, it suggests that quantitative CT-derived attenuation parameters and metabolic indices may provide complementary information regarding hepatic involvement in patients with adrenal incidentaloma, potentially reflecting different aspects and stages of the disease process.

This study has several strengths. To our knowledge, it is among the few studies to comprehensively evaluate CT-derived hepatic attenuation parameters in patients with adrenal incidentaloma while simultaneously comparing conventional CT-defined steatosis thresholds with multiple validated metabolic indices, including TyG, TyG-WHtR, METS-IR, FLI, and FIB-4. Furthermore, by examining several markers of cortisol secretion, including post-DST cortisol, ACTH, midnight serum cortisol, and 24-h UFC, we were able to explore the relationship between different aspects of cortisol excess and hepatic fat accumulation. This comprehensive approach provides additional insight into the complex interaction between mild cortisol excess and hepatic steatosis.

Several limitations should be acknowledged. First, the retrospective cross-sectional design precludes any conclusions regarding causality between cortisol excess and hepatic fat accumulation. Second, the study was conducted at a single tertiary referral center with a relatively limited sample size, particularly in the MACS subgroup, which may have reduced statistical power for some analyses. Third, hepatic steatosis was assessed using unenhanced CT attenuation measurements rather than magnetic resonance imaging or histological evaluation. Although CT-derived attenuation parameters are widely accepted and readily available in routine clinical practice, they are less sensitive than advanced quantitative imaging techniques for detecting mild steatosis. Finally, longitudinal follow-up was not available; therefore, the prognostic significance of subtle changes in hepatic attenuation could not be determined.

## 5. Conclusions

In conclusion, patients with MACS exhibit subtle CT-derived hepatic fat accumulation despite similar conventional CT-defined steatosis and non-invasive metabolic indices compared with patients with NFAI. Continuous CT-derived attenuation parameters may provide additional information for detecting subtle hepatic differences associated with mild cortisol excess. Among patients with MACS, 24-h urinary free cortisol showed consistent inverse associations with CT-derived hepatic attenuation parameters, including a significant association with liver–spleen attenuation difference after adjustment for age, sex, and BMI. These findings warrant confirmation in larger prospective studies.

## Figures and Tables

**Table 1:** Baseline characteristics of patients in NFAI and MACS groups.

Characteristic	NFAI (*n* = 84)	MACS (*n* = 39)	*p* Value
**Clinical characteristics**			
Age, years	56.4 ± 10.9	54.0 ± 9.6	0.702
Female sex, *n* (%)	62 (73.8)	28 (71.8)	0.814
Body mass index, kg/m^2^	30.2 ± 4.7	31.0 ± 5.9	0.389
Hypertension, *n* (%)	37 (44.0)	21 (53.8)	0.311
Diabetes mellitus, *n* (%)	27 (32.1)	16 (41.0)	0.336
Dyslipidemia, *n* (%)	16 (19.0)	14 (35.9)	**0.043**
**Adrenal characteristics**			
Maximum adenoma diameter, mm	20.3 ± 8.3	30.5 ± 11.0	**<0.001**
Unenhanced adenoma attenuation, HU	−5.1 (−11.7 to 5.0)	−3.6 (−12.6 to 6.7)	0.875
Lesion laterality, *n* (%)			**0.042**
• Right	32 (38.1)	13 (33.3)	
• Left	35 (41.7)	10 (25.6)	
• Bilateral	17 (20.2)	16 (41.0)	
**Hormonal parameters**			
Post-DST cortisol, µg/dL (< 1.8)	1.00 (0.70–1.35)	2.30 (2.02–4.00)	**<0.001**
Basal ACTH, pg/mL (10–60)	15.35 (11.05–21.95), *n* =70	10.55 (7.46–13.75), *n* = 39	**0.001**
24-h urinary free cortisol, µg/24 h	165.8 (105.0–233.1), *n* =31	214.1 (146.7–264.0), *n* =36	0.071
**Metabolic and biochemical parameters**			
Fasting glucose, mg/dL (70–99)	99 (91–110)	93.5 (86.5–112.0)	0.351
HbA1c, % (<5.7)	5.8 (5.55–6.60)	6.0 (5.70–6.40)	0.433
Triglycerides, mg/dL (<150)	142.5 (93.0–189.0)	138.5 (103.5–193.0)	0.113
HDL cholesterol, mg/dL (women ≥50; men ≥40)	58.5 ± 12.4	54.4 ± 11.8	**0.039**
LDL cholesterol, mg/dL (<100)	124.9 ± 29.0	133.3 ± 28.9	0.295
AST, U/L (10–40)	17.5 (14.5–22.0)	18.0 (15.0–19.5)	0.445
ALT, U/L (7–56)	16.0 (13.0–21.5)	13.0 (11.0–20.5)	0.118
GGT, U/L (women 5–36; men 8–61)	24.5 (18.0–29.5)	22.0 (18.0–28.0)	0.715
Platelet count (150–450 × 10^9^/L)	269.8 ± 70.1	260.6 ± 69.7	0.206

Footnote: Continuous data are summarized as means ± SDs or medians (25th–75th percentiles), depending on their distribution; categorical data are reported as *n* (%). Between-group comparisons used the independent-samples t-test or Mann–Whitney U test for continuous variables and Pearson’s χ^2^ test or Fisher’s exact test for categorical variables, as appropriate. Abbreviations: ACTH, adrenocorticotropic hormone; ALT, alanine aminotransferase; AST, aspartate aminotransferase; DST, dexamethasone suppression test; GGT, gamma-glutamyl transferase; HbA1c, glycated hemoglobin; HDL, high-density lipoprotein; HU, Hounsfield unit; LDL, low-density lipoprotein; MACS, mild autonomous cortisol secretion; NFAI, non-functioning adrenal incidentaloma; SD, standard deviation.

**Table 2:** CT-based hepatic steatosis parameters and non-invasive hepatic steatosis/fibrosis scores in patients with NFAI and MACS patients.

Characteristic	NFAI (*n* = 84)	MACS (*n* = 39)	*p* Value
**CT-based hepatic steatosis parameters**			
Waist circumference on CT, cm	102.0 (93.1–109.9)	103.6 (99.2–107.7)	0.870
Splenic attenuation, HU	55.0 ± 5.2	54.5 ± 5.5	0.274
Hepatic attenuation, HU	65.8 (60.2–72.5)	63.5 (59.5–67.9)	0.167
Liver-to-spleen attenuation ratio	1.24 (1.06–1.37)	1.15 (1.07–1.30)	**0.050**
Liver–spleen attenuation difference, HU	13.2 (3.2–18.8)	8.2 (3.8–15.5)	**0.048**
Hepatic attenuation <40 HU, *n* (%)	3 (3.6)	2 (5.1)	0.684
Liver-to-spleen ratio <1.0, *n* (%)	13 (15.5)	6 (15.4)	0.990
Liver–spleen difference <5 HU, *n* (%)	18 (21.4)	11 (28.2)	0.410
**Non-invasive hepatic steatosis/fibrosis scores**			
FIB-4 index	0.75 (0.65–1.01)	0.84 (0.64–1.14)	0.251
Fatty Liver Index (FLI)	54.2 (33.4–79.6)	74.4 (56.6–84.1)	0.176
TyG index	8.63 ± 0.62	9.09 ± 0.55	0.075
Waist-to-height ratio	0.64 (0.57–0.69)	0.64 (0.58–0.68)	0.528
TyG-WHtR	5.48 ± 0.85	5.65 ± 0.83	0.085
METS-IR	44.1 ± 8.0	45.3 ± 7.0	0.067

Footnote: Values are reported as means ± SDs, medians (25th–75th percentiles), or counts (%). Differences between groups were assessed with the independent-samples t-test, Mann–Whitney U test, Pearson’s χ^2^ test, or Fisher’s exact test, as appropriate. Abbreviations: CT, computed tomography; FIB-4, Fibrosis-4 index; FLI, Fatty Liver Index; HU, Hounsfield unit; MACS, mild autonomous cortisol secretion; METS-IR, metabolic score for insulin resistance; NAFLD, nonalcoholic fatty liver disease; NFAI, non-functioning adrenal incidentaloma; TyG, triglyceride-glucose index; TyG-WHtR, triglyceride-glucose waist-to-height ratio.

**Table 3:** Correlations between cortisol-axis parameters and CT-derived hepatic steatosis parameters in patients with MACS.

Cortisol Parameter	Liver Attenuation (HU)	Liver-to-Spleen Ratio	Liver-Spleen Attenuation Difference
Post-DST cortisol	ρ = −0.012, *p* = 0.940	ρ = 0.151, *p* = 0.360	ρ = 0.115, *p* = 0.485
Basal ACTH	ρ = −0.006, *p* = 0.971	ρ = −0.061, *p* = 0.713	ρ = −0.056, *p* = 0.733
Late-night cortisol	ρ = −0.184, *p* = 0.269	ρ = −0.042, *p* = 0.802	ρ = −0.046, *p* = 0.783
24-h urinary free cortisol	**ρ = −0.427, *p* = 0.009**	**ρ = −0.370, *p* = 0.026**	**ρ = −0.396, *p* = 0.017**

Footnote: Data are presented as Spearman’s rank correlation coefficients (ρ) and corresponding *p* values. Correlation analyses were performed in the 36 patients in the MACS subgroup with available 24-h urinary free cortisol measurements. Abbreviations: ACTH, adrenocorticotropic hormone; DST, dexamethasone suppression test; HU, Hounsfield unit; MACS, mild autonomous cortisol secretion.

**Table 4:** Multivariable linear regression analyses of CT-derived hepatic steatosis parameters in patients with mild autonomous cortisol secretion (MACS).

Variable	Liver Attenuation (HU)	Liver-to-Spleen Attenuation Ratio	Liver-Spleen Attenuation Difference (HU)
BMI	**β = −0.365, *p* = 0.038**	β = −0.098, *p* = 0.590	β = −0.075, *p* = 0.680
UFC	β = −0.362, *p* = 0.056	β = −0.397, *p* = 0.051	**β = −0.403, *p* = 0.047**
Age	β = −0.103, *p* = 0.569	β = −0.200, *p* = 0.301	β = −0.222, *p* = 0.252
Sex	β = 0.105, *p* = 0.612	β = 0.160, *p* = 0.472	β = 0.165, *p* = 0.457
Adjusted R^2^	0.153	0.032	0.034
Overall model P	0.057	0.295	0.288

Footnote: Data are presented as standardized regression coefficients (β) and corresponding *p* values. Separate multivariable linear regression models were constructed for each CT-derived hepatic steatosis parameter using the Enter method. Each model included age, sex, body mass index, and urinary free cortisol as independent variables. All regression analyses were performed in the 36 patients in the MACS subgroup with available 24-h urinary free cortisol measurements. Abbreviations: BMI, body mass index; HU, Hounsfield unit; UFC, urinary free cortisol.

## Data Availability

Data supporting the study findings can be obtained from the corresponding author on reasonable request. Public access is restricted to protect participants’ privacy, as the dataset includes potentially identifying information.

## Abbreviations

The following abbreviations are used in this manuscript:

AI	Adrenal incidentaloma
ACTH	Adrenocorticotropic hormone
BMI	Body mass index
CT	Computed tomography
DST	Dexamethasone suppression test
FIB-4	Fibrosis-4 index
FLI	Fatty Liver Index
HU	Hounsfield unit
L/S ratio	Liver-to-spleen attenuation ratio
L–S difference	Liver–spleen attenuation difference
MACS	Mild autonomous cortisol secretion
MASLD	Metabolic dysfunction-associated steatotic liver disease
METS-IR	Metabolic Score for Insulin Resistance
NFAI	Non-functioning adrenal incidentaloma
TyG	Triglyceride-glucose index
TyG-WHtR	Triglyceride-glucose waist-to-height ratio
UFC	24-h urinary free cortisol
